# Interparental Relationship Discord and Adolescent Psychopathology in a United States Probability Sample

**DOI:** 10.1007/s10802-025-01289-y

**Published:** 2025-01-31

**Authors:** Elisa F. Stern, Soo Hyun Rhee, Mark A. Whisman

**Affiliations:** Department of Psychology and Neuroscience, University of Colorado Boulder, 345 UCB, Boulder, CO 80309-0345 USA

**Keywords:** Adolescent, Interparental discord, Parent, Psychopathology, Mental disorder

## Abstract

Exposure to interparental conflict and poor parental relationship adjustment (i.e., interparental relationship discord) has been associated with children’s internalizing and externalizing symptoms throughout childhood and later life. However, the degree to which interparental relationship discord is associated with clinical levels of psychopathology in adolescents remains unclear. The association between parents’ report of interparental relationship discord and mental disorders in adolescents was investigated in the National Comorbidity Survey–Adolescent Supplement, a United States probability sample of 13-17-year-old adolescents and their parents (*N* = 4,112 dyads). A hierarchical framework, consisting of 16 specific disorders, latent dimensions of internalizing and externalizing disorders, and a latent dimension of general psychopathology, was employed. Greater interparental relationship discord demonstrated small but significant associations with higher levels of general psychopathology and internalizing and externalizing psychopathology, as well as with several specific disorders. Overall, results are consistent with the perspective that interparental relationship discord may increase risk for clinical levels of psychopathology in adolescents.

Poor parental relationship functioning is a robust predictor of children’s adjustment across different domains of functioning (i.e., social, emotional, behavioral, cognitive, and physiological) (for reviews, see Harold & Sellers, 2018; van Eldik et al., 2020). The present study was conducted to evaluate the degree to which interparental relationship discord is associated with clinically significant levels of psychopathology during adolescence in a nationally representative sample from the United States.

In studying interparental relationships and offspring psychopathology, researchers have examined a variety of facets of parents’ relationship functioning. Some researchers have focused exclusively on interparental conflict, seeking to identify domains of conflict (e.g., frequency, intensity, or type of conflict) that are most strongly associated with offspring’s mental health (van Eldik et al., 2020). In comparison, other researchers have adopted a broader focus of the parents’ relationship and have examined the association between the quality of parents’ relationship and offspring psychopathology. Like much of the literature on the quality of intimate relationships, there are a variety of terms that are used to characterize the construct measured in these latter studies, including relationship satisfaction, quality, and adjustment, to name a few. Fincham and Rogge (2010) recommended distinguishing between measures of *relationship satisfaction* (or *quality*), which include only subjective evaluations of the relationship, from measures of *relationship adjustment*, which typically include interpersonal processes such as communication and companionship, as well as subjective evaluations. Measures of relationship adjustment typically include items assessing conflict (i.e., items assessing the frequency of how often partners disagree about topics in their relationship), as well as other interpersonal processes and subjective evaluations of the relationship. We follow Fincham and Rogge’s distinction in this paper, using the term interparental relationship adjustment to refer to this broader construct that includes but is not limited to frequency of conflict, to align this work within the broader literature on relationship functioning. Furthermore, because we focused on poor parental relationship adjustment as a risk factor for psychopathology, we refer to parents’ relationship adjustment in terms of *interparental relationship discord* (rather than low interparental relationship adjustment), with higher levels of discord hypothesized to be associated with increased risk for offspring psychopathology. This is like the conceptual approach used by van Eldik et al. (2020) in their meta-analysis of interparental relationships and children’s maladjustment discussed below.

## Interparental Conflict and Child Psychopathology

A robust literature has indicated that children exposed to several dimensions of interparental conflict (i.e. frequency, severity, resolution) are at an increased risk for developing a variety of adverse outcomes, including various forms of psychopathology, aggression, poor academic attainment, physical health problems, and worsened future intimate relationship adjustment (Bernet et al., 2016; Cummings & Davies, 2002; Harold & Sellers, 2018; Rhoades, 2008). More specifically, conflict exposure is associated with both (a) externalizing behaviors, including elevated symptoms of aggression, conduct related problems, and antisocial behaviors that begin in childhood and persist across adolescence and young adulthood (Davies et al., 2016; Harold & Sellers, 2018); and (b) internalizing behaviors, including symptoms consistent with depression, anxiety, low self-esteem, and when extreme, suicidality (Harold & Sellers, 2018).

## Interparental Relationship Discord and Child Psychopathology

Research on interparental relationship functioning and child psychopathology has tended to focus on interparental conflict, rather than interparental relationship adjustment or discord, with the assumption that overt conflict that occurs in front of children poses a greater risk for adverse child outcomes (Davies & Cummings, 1994; Grych & Fincham, 1990). However, a recent meta-analysis found that interparental relationship discord (discussed as relationship quality in the study) was as strongly associated with children’s internalizing (i.e. depressive or anxious behaviors) and externalizing (i.e. aggression or delinquent behaviors) psychopathology as interparental conflict (van Eldik et al., 2020). As outlined above, interparental relationship functioning extends beyond conflict alone, incorporating dimensions of satisfaction, cohesion, affection, and consensus on matters of importance to dyadic functioning (Spanier, 1976; van Eldik et al., 2020). Prior works have suggested that non-conflict specific factors, such as parental distress and negativity, can spill over to parent–child relationships and/or may lead to disrupted family boundaries (i.e., children’s parentification), influencing children’s emotion regulation and expression negatively over time (Nuttall et al., 2017; van Eldik et al., 2020). As noted elsewhere (e.g., Cui et al., 2005), whereas overt conflict that occurs in front of children may be more salient for younger children, adolescents may be more aware of and sensitive to more subtle expressions of intimacy, affection, and communication characteristic of relationship discord.

These findings underscore the importance of considering a broader conceptualization of the interparental relationship when trying to understand its association with developmental psychpathology. In Van Eldik and colleagues’ (2020) meta-analysis, 10 studies examined this association in exclusively adolescent samples. Of these 10 studies, one study was based on a sample of people with motor and intellectual disabilities and therefore may have limited generalizability (Vrijmoeth et al., 2012). Of the nine remaining studies, only five examined both internalizing and externalizing psychopathology. Of those five, three examined only one internalizing and externalizing outcome (David et al., 1996; Feinberg et al., 2007, Peris & Emery., 2004). Feinberg and colleagues (2007), for example, found significant cross-sectional positive associations between interparental relationship discord and adolescent depression and antisocial behavior, whereas Peris and Emery (2004) found associations between interparental relationship discord and adolescent internalizing symptoms (measured by a single “feelings” scale) and delinquency. Given the small number of studies that have examined interparental relationship discord and multiple indicators of internalizing and externalizing psychopathology in adolescents, particularly in terms of latent dimensions of internalizing and externalizing psychopathology, there is a need for additional research examining these associations.

## Adolescent Psychopathology as Mental Disorders, Rather than Symptom-Based Dimensions

Thus far, the association between interparental relationship functioning and psychopathology in children has been most profiled using a dimensional taxonomy of symptoms, operationalized using externalizing and internalizing domains. What remains less clear is the degree to which interparental conflict and relationship discord are associated with clinically significant levels of psychopathology throughout adolescence. The diagnosis of a mental disorder requires that a person is experiencing symptoms with sufficient frequency or severity to result in distress or impairment (American Psychiatric Association, 2022). Clarifying the potential influence of interparental relationship functioning on clinical levels of psychopathology is particularly salient during adolescence, given that most mental health conditions are thought to onset during this period (Solmi et al., 2022).

Of the nine studies that involved only adolescent samples in the van Eldik et al. (2020) meta-analysis, only one operationalized psychopathology in terms of mental disorders (Hammen et al., 2004). However, this study examined the association between adolescent psychopathology (depression, externalizing disorders) and the interaction between maternal relationship satisfaction and maternal depression status and did not report on the bivariate association between maternal relationship satisfaction and adolescent psychopathology. As such, the existing literature has not fully evaluated the extent to which components of parents’ relationships are associated with more severe presentations of psychopathology in adolescents.

Given that previous studies have examined psychopathology in terms of symptom domains, there is merit in assessing whether these findings are consistent when measured as latent factors derived from diagnoses, rather than symptoms. Studies examining the latent structure of mental disorders in children and adults have found that several factors represent predispositions to internalizing or externalizing psychopathology that aptly capture the co-occurrence of different mental disorders. For example, Blanco et al. (2015) examined the factor structure of 16 mental disorders in a United States probability sample and found evidence for two correlated second-order factors measuring internalizing and externalizing psychopathology, as well as a third-order factor measuring general psychopathology.

## Present Study

The aim of the current study was to build upon existing literature indicating that interparental relationship discord is associated with broad dimensions of psychopathology in children and adolescents, constructed by individual or clustered symptoms, by evaluating the degree to which this association is found for clinically significant levels of psychopathology during adolescence. We first examined the association between interparental relationship discord and adolescent psychopathology using the Blanco et al. (2015) hierarchical framework, consisting of (a) second-order latent dimensions of internalizing and externalizing disorders derived from diagnoses for 16 mental disorders, and (b) a third-order latent dimension of general psychopathology (Blanco et al., 2015). We then examined the association between interparental relationship discord and each of the 16 disorders separately to identify which are associated with interparental relationship discord. In comparison to prior studies that have operationalized adolescent psychopathology in terms of symptoms, sometimes based on a single component of internalizing or externalizing psychopathology, this approach involves a more comprehensive assessment of internalizing and externalizing psychopathology. It has the potential to add crucial information about how interparental relationship discord is associated with clinical levels of psychopathology measured in terms of (a) latent dimensions of internalizing, externalizing, and general psychopathology, based on mental disorders; and (b) specific mental disorders. Findings regarding higher-order dimensions (i.e., internalizing and externalizing latent factors) may be most relevant for etiological research whereas findings regarding specific diagnoses may be more useful for clinicians to aid in their treatment decision-making for individual patients (Blanco et al., 2015). Additionally, clarifying the potential association between interparental relationship discord and psychopathology during adolescence may lend itself well to early detection and intervention, given this developmental period is regarded as a time of burgeoning psychopathology (McGorry & Mei, 2018).

## Method

### Participants

The present study involved secondary analysis of data from 4,112 adolescents and their parents from the National Comorbidity Survey–Adolescent Supplement (NCS-A; Kessler, 2023), a United States probability sample of adolescents aged 13–17 years. It includes a household and school sample from a representative sample of schools in the catchment area (Kessler et al., 2009); the overall adolescent response rate was 82.9%. There were 4,602 adolescents whose parents were either currently married or living with someone in a marriage-like relationship. Parents who were married or cohabiting for at least one year (i.e., the time frame for the assessment of adolescent psychopathology) were included; data from parents who had been married < 1 year (*n* = 95) or who did not provide data on length of their current relationship (*n* = 223) were excluded. Because the focus of this study was on interparental relationship discord, we excluded data from non-nuclear parent/caregivers (e.g., grandparents) (*n* = 123). Finally, data from parents were excluded if they missed ³ 4 items (³ 29%) on the measure of interparental relationship discord (*n* = 49). The sample of adolescents was 51% female (specified by binary sex), and the racial/ethnic distribution was 74% White, 10% Black, 10% Latino, and 6% other; adolescents had a mean age of 15.1 years (*SD* = 1.4 years). The sample of parents was 89% female and 96% of the parents were biological parents of the adolescent; most parents were married (93%) and the average length of the relationship between parents was 17.7 years (*SD* = 7.3 years; range = 1–42 years).

Because the study involved a secondary analysis of deidentified data and was therefore not human subjects research, it was exempt from Institutional Review Board review.

### Measures

#### Adolescent Psychopathology

Adolescents were interviewed with the Composite International Diagnostic Interview (CIDI), modified to simplify language, and using examples relevant to adolescents (Merikangas et al., 2009). Included were *Diagnostic and Statistical Manual of Mental Disorders*,* Fourth Edition* diagnoses of mood disorders (major depressive episode [MDE], dysthymic disorder, bipolar I disorder, bipolar II disorder), anxiety disorders (panic disorder, agoraphobia, social phobia, specific phobia, generalized anxiety disorder [GAD], post-traumatic stress disorder [PTSD], separation anxiety disorder), eating disorders (anorexia, bulimia, binge eating disorder), behavioral disorders (attention-deficit/hyperactivity disorder [ADHD], oppositional defiant disorder, conduct disorder), and substance use disorders (alcohol abuse/dependence, drug abuse/dependence, nicotine dependence). Parents completed a self-administered questionnaire developed for the NCS-A that assessed several of these disorders (Merikangas et al., 2009). Consistent with studies using data from other samples (Bornovalova et al., 2014; Feinberg et al., 2007), and other studies using the NCS-A data (e.g., Kessler et al., 2012; Merikangas et al., 2010), adolescent and parent reports were combined at the symptom level using an “or” rule (i.e., a symptom was coded as present if endorsed by either adolescent or parent) for depressive disorders and behavior disorders to improve validity, as prior research has shown that parent reports are particularly important for making these diagnoses (Grills & Ollendick, 2002). In comparison, only parent reports were used for attention-deficit/hypeactivity, similar to other studies using the the NCS-A data (Kessler et al., 2012), and the remaining disorders were based on adolescents’ report only. Strong concordance was found between CIDI diagnosis (based on adolescent, parent, or both adolescent and parent report as described above) and clinical reassessments made using the Schedule for Affective Disorders and Schizophrenia for School-Age Children Lifetime Version (Kessler et al., 2009).

##### Interparental Relationship Discord

Parents’ report of interparental relationship discord was measured with the Dyadic Adjustment Scale (DAS; Spanier, 1976), which was modified for use in the NCS-R (Whisman, 2007). Specifically, the NCS-R included 14 items (1, 2, 5, 8, 12, 16, 18, 20, 21, 24, 25, 26, 27, and 28) from the DAS, The scaling and response options were modified from the original DAS: 9 items rated on a 6-point scale in the original DAS were rated on a 5-point scale in the NCS-R, four items rated on a 6-point scale in the original DAS were rated on a 4-point scale in the NCS-R, and one item rated on a 5-point scale in the original DAS was rated on a 4-point scale in the NCS-R. Items were recoded so that higher scores indicate greater relationship discord; expectation-maximization was used to impute values for missing item data for parents who were missing data on < 4 items. Items were standardized and averaged to create a composite scale (standardized McDonald’s omega [ω] = 0.90), and a constant was added so that the minimum score was 0.

### Statistical Analyses

**Fig. 1:**
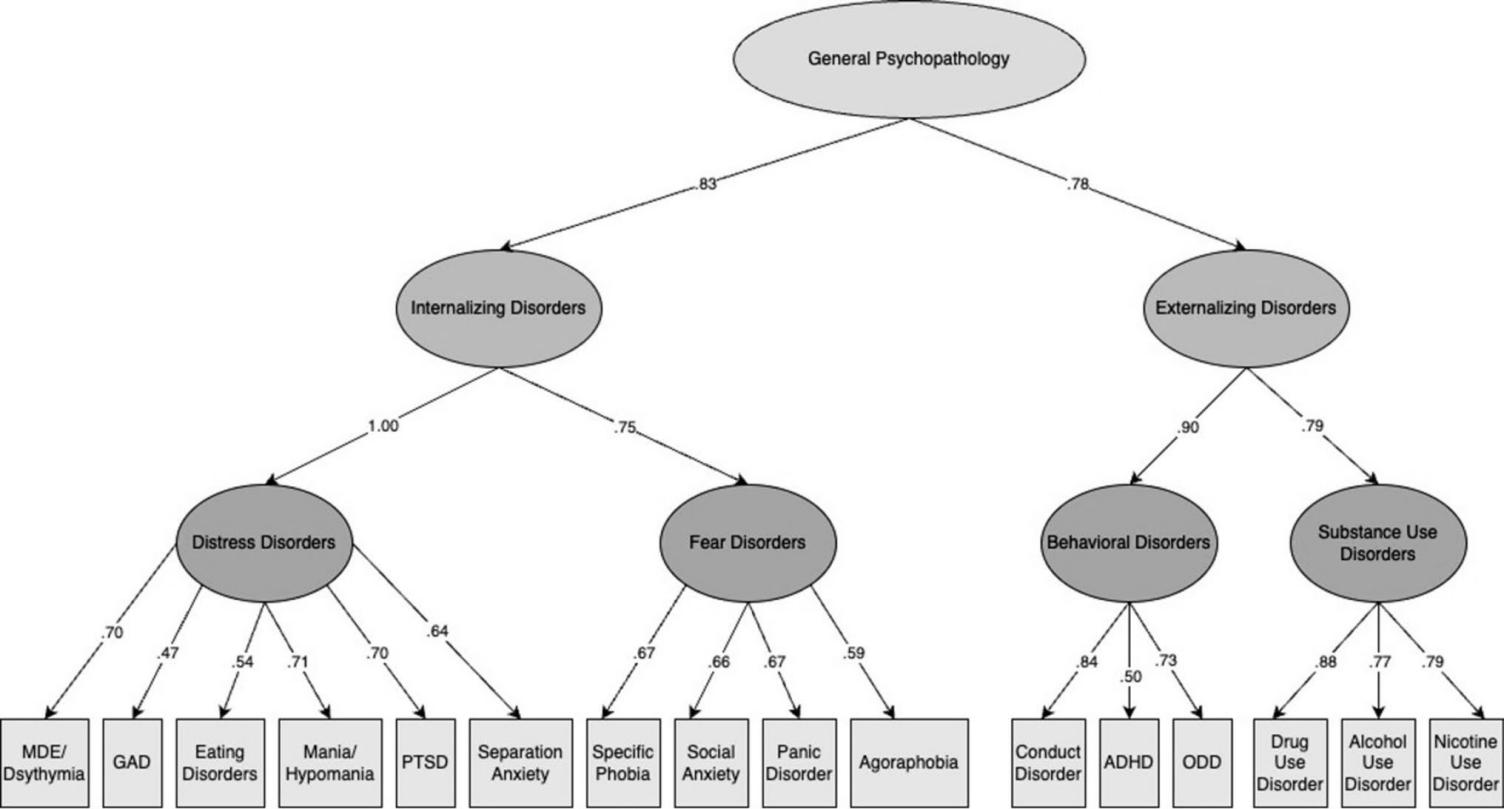
Hierarchical factor structure of common mental disorders in adolescence.Originally reported and tested in Blanco et al. (2015) *Note.* MDE = major depressive episode; GAD = generalized anxiety disorder, PTSD = post-traumatic stress disorder; ADHD = attention-deficit hyperactivity disorder; ODD = oppositional defiant disorder. Residual variance of the Distress Disorders latent factor was fixed to be 0. Standardized parameters are presented

The first operationalization of adolescent psychopathology was based on the hierarchical latent factor structure obtained by Blanco et al. (2015) based on 12-month prevalence data for mental disorders in the NCS-A. We tested a hierarchical model with a third-order general psychopathology factor, with loadings on second-order internalizing and externalizing factors, which in turn had loadings on first-order distress disorders, fear disorders, behavioral disorders, and substance use disorders, which in turn had loadings on specific mental disorders (see Fig. 1). We examined the covariation between parents’ report of interparental relationship discord and (a) the general psychopathology factor, and (b) the internalizing and externalizing factors. Analyses were conducted in MPlus Version 8.0 (Muthén & Muthén, 1998–2017) using weighted least square mean and variance adjusted (WLSMV) estimation.

To examine the association between interparental discord and 16 specific disorders, logistic regression analyses were then conducted in which the 12-month prevalence of each disorder was regressed on parents’ report of interparental discord. The exponential of the logistic coefficient from each analysis was computed and expressed as an odds ratio (OR); the 95% confidence interval (95% CI) for each odds ratio was also calculated. Dividing the logistic coefficient by p/√3 results in a logit *d* that is comparable to the familiar standardized mean difference statistic involving two groups (i.e., *d*), which is presented as a measure of effect size.

## Results

The 12-month prevalence for each of the 16 disorders are presented in Table 1.

**Table 1:** Prevalence of mental disorders and associations with interparental relationship discord

Disorder	Prevalence	Association with Relationship Discord		
	(%)	*OR*	95% *CI*	*d* ^a^
	Internalizing Psychopathology			
*Distress Disorders*				
MDE/Dysthymia	7.6	1.27**	1.08, 1.49	0.13
Bipolar I/Bipolar II	2.0	1.11	0.81, 1.53	0.06
Eating Disorder	2.8	1.22	0.93, 1.59	0.11
GAD	1.0	1.35	0.89, 2.03	0.16
PTSD	2.4	1.06	0.79, 1.43	0.03
Separation Anxiety Disorder	1.0	1.13	0.73, 1.75	0.07
*Fear Disorders*				
Panic Disorder	1.9	1.04	0.74, 1.46	0.02
Agoraphobia	1.1	1.37	0.91, 2.05	0.17
Social Phobia	11.2	1.28***	1.11, 1.47	0.14
Specific Phobia	13.5	1.14	1.00, 1.29	0.07
	Externalizing Psychopathology			
*Behavioral Disorders*				
ADHD	6.4	1.38***	1.16, 1.64	0.18
Oppositional Defiant Disorder	4.2	1.28*	1.03, 1.58	0.13
Conduct Disorder	6.3	1.72***	1.46, 2.03	0.30
*Substance Use Disorders*				
Alcohol Use Disorder	4.4	1.35**	1.10, 1.66	0.16
Drug Use Disorder	4.8	1.27*	1.04, 1.55	0.13
Nicotine Dependence	4.8	1.28*	1.05, 1.57	0.14

^a^Logit *d* values. *MDE * major depressive episode; *GAD* generalized anxiety disorder, *PTSD * post-traumatic stress disorder; *ADHD* attention-deficit hyperactivity disorder

* *p* < .05. ** *p* < .01. *** *p* < .001

The weighted mean level of interparental relationship discord was 1.18 (*SD* = 0.66; range, 0.00–4.45).

Factor loadings for the hierarchical model of adolescent psychopathology can be found Fig. 1. Fit indices indicate that the model fit the data well, χ^2^(100) = 226.79, *p* < .001, Comparative Fit Index (CFI) = 0.96, Root Mean Square Error of Approximation (RMSEA) = 0.02; CFI values ≥ 0.95 and RMSEA values ≤ 0.06 are viewed as evidence for a well-fitting model (Hu & Bentler, 1999). The correlated factors model, which had the same fit statistics, indicated a correlation of 0.65, *p* < .001, between the internalizing and externalizing factor.

The correlation between interparental relationship discord and adolescent general psychopathology was 0.16, *p* < .001. The association between interparental relationship discord and the externalizing factor (*r* = .17, *p* < .001) was significantly greater than that with the internalizing factor (*r =* .09, *p* < .001), *t*(4109) = 3.69, *p* < .001.

As can be seen in Table 1, compared to parents of adolescents without the corresponding disorder, greater interparental relationship discord was reported by parents of adolescents who met criteria for two of the assessed internalizing disorders (MDE/dysthymia, social phobia) and each of the assessed externalizing disorders (ADHD, oppositional defiant disorder, conduct disorder, alcohol use disorder, drug use disorder, and nicotine dependence). ORs presented in Table 1 indicate the change in odds for meeting diagnostic criteria for the disorder with a one-unit (i.e., one-point) change in interparental relationship discord. For example, the OR of 1.27 for MDE/dysthymia indicates that a one-point increase in interparental relationship discord was associated with a 27% increase in the odds of meeting diagnostic criteria for MDE or dysthymia.

## Discussion

The first purpose of this study was to evaluate, in a probability sample of American 13–17-year-old adolescents, the association between interparental relationship discord and adolescent psychopathology using a hierarchical framework for assessing psychopathology based on diagnoses of mental disorders. Interparental relationship discord demonstrated a small but significant association with higher levels of a latent factor measuring general psychopathology and with latent factors measuring internalizing and externalizing disorders. The effect sizes observed in current study are similar in magnitude or larger than those reported in prior research (0.15 for externalizing behavior and 0.12 for internalizing behavior in van Eldik et al.’s [2020] meta-analysis). Importantly, most of the studies in the meta-analyses focused on symptoms rather than diagnoses and on children rather than adolescents. In the present study, interparental relationship discord had a stronger association with the externalizing latent factor relative to the internalizing latent factor. This finding is important insofar as prior research has generally found that dimensions of the interparental relationship are generally not more strongly associated with some domains of child functioning than with other domains (van Eldik et al., 2020). Because most of these studies have examined symptoms whereas we examined disorders, it may be that the stronger association between interparental relationship discord and externalizing psychopathology in adolescents is obtained at the level of psychopathology more often found in clinical settings. Previous research also suggests that overall, clinically significant externalizing disorders (e.g., ADHD, CD) onset earlier than internalizing disorders (e.g., GAD, PTSD, mood disorder) (Kessler et al., 2007). Although it is possible that the full extent of psychopathology had not onset in our sample at the time of data collection, it awaits further investigation to evaluate whether the pattern of results obtained in the present study replicates in other studies using latent factors of internalizing and externalizing psychopathology based on diagnoses of mental disorders.

The second purpose of the study was to examine the association between interparental relationship discord and specific mental disorders. Interparental relationship discord was significantly and positively associated with two of the assessed internalizing disorders (MDE/dysthymia, social phobia) and each of the assessed externalizing disorders (ADHD, oppositional defiant disorder, conduct disorder, alcohol use disorder, drug use disorder, and nicotine dependence) (see Table 1). The association between interparental relationship discord and several other mental disorders (e.g., GAD, agoraphobia), although not statistically significant, yielded meaningful effect sizes that were comparable in magnitude to some of the associations that were statistically significant. Because these other disorders had lower base rates, it is likely that these associations were not statistically significant due to lower statistical power. Overall, the current findings are noteworthy, as they suggest that interparental relationship discord is associated with clinical levels of psychopathology in adolescents, as measured in terms of latent dimensions of internalizing, externalizing, and general psychopathology, as well as measured in terms of several specific mental disorders. These findings are consistent with the perspective that interparental relationship discord may increase risk for a variety of mental health problems in adolescents (Yap & Jorm, 2015).

Additional research is needed to increase understanding of the mechanisms explaining the association between interparental relationship discord and adolescent psychopathology. Much of the research on the association between parental relationship functioning and psychopathology in children and adolescents is based on the cognitive-contextual framework (Grych & Fincham, 1990) and the emotional security hypothesis (Davies & Cummings, 1994) theoretical models. The cognitive-contextual framework posits that the impact of interparental conflict and discord on psychopathology in children is mediated by children’s negative responses to the conflict and discord, including the extent to which they feel threatened by and make self-blaming appraisals, which are impacted by contextual, cognitive, and developmental factors. In comparison, the emotional security hypothesis proposes that interparental conflict and discord influence children’s emotion regulation, cognitive representations, and behavioral regulation, which in turn influence their sense of emotional security. Over time, this threatened sense of emotional security can lead to longer-term adjustment problems. Whereas overt interparental conflict may need to occur in front of children for these processes to be activated for younger children, more subtle expressions of intimacy, affection, and communication characteristic of interparental relationship discord may be sufficient to activate these processes in adolescents (cf. Cui et al., 2005). In van Eldik et al.’s (2020) meta-analysis, there were less than three studies examining the association between interparental relationship discord and emotional, behavioral, physiological, and emotional insecurity outcomes that may serve as putative mechanisms. Thus, they were unable to conduct a meta-analysis or compute meaningful effect sizes for these outcomes.

An important topic for future research is evaluating the multiple pathways by which parental psychopathology, interparental relationship discord, and adolescent psychopathology are associated. For example, given the well-established association between relationship discord and psychopathology in partnered individuals (South, 2021), particularly with respect to depression (Whisman et al., 2021), interparental relationship discord may increase the likelihood of parental psychopathology, which may in turn increase the probability of adolescent psychopathology. In support of this perspective, maternal psychopathology (Davies et al., 1999) and parental psychopathology (Papp et al., 2004) have been shown to mediate the association between interparental relationship discord and psychopathology in adolescents and children, respectively. On the other hand, parental psychopathology in either parent may increase interparental relationship discord, which in turn influences adolescent psychopathology (Hanington et al., 2012). Research evaluating interparental relationship discord as a mediator in the association between parental and adolescent psychopathology is also needed.

Results from the current study should be interpreted considering several limitations. Most of these limitations—several stemming from the data available in the NCS-R—are related to issues of generalizability and interpretability. Below we discuss each limitation in detail and provide recommendations for future research.

First, ratings of relationship discord were completed primarily by the biological mother of the adolescent (89% mother-report; 96% biological parent). Additionally, most children in this study had intact families (93% of parents were married). It is possible that these characteristics influenced reports of relationship discord and/or reports of adolescent’s mental health (for the disorders in which parental report was included). For example, mothers and fathers may differ in their assessment of their relationship, and thus the lack of father-reported relationship discord should be considered in interpreting the present findings. Additionally, the generalizability of our study findings to non-traditional family structures may be limited. For example, prior literature has suggested that parental divorce and interparental relationship discord are associated with different psychosocial outcomes in adolescents (Amato & Sobolewski, 2001; Aro, 1988). As such, replication of these analyses in diverse families, such as in divorced, blended, same-sex, and adoptive families, with both parents reporting concurrent relationship discord to the extent possible, is an important next step. Importantly, although the assessment of interparental relationship discord was limited to parent report (versus adolescent perceptions of their parents’ discord), previous research has demonstrated that parents’ self-reported relationship discord (e.g., DAS scores) is significantly associated with both (a) adolescents’ perceptions of their parents’ relationship discord, as measured by the DAS (Davern et al., 2005); and (b) adolescents’ reports of interparental conflict (Wymbs et al., 2008). This is notable, particularly given the cognitive-contextual framework suggests that adolescents’ perception of their parents’ relationship discord is a primary contributor to symptoms of psychopathology (Grych & Fincham, 1990).

Second, despite the large sample size overall, the base rates for specific disorders were low. As such, the study was underpowered to test binary sex as a potential moderator of the association between interparental relationship discord and adolescent psychopathology. This is potentially problematic, given several studies have found that the association between other risk factors (e.g. COVID-19) and the emergence of psychopathology can be moderated by sex (Curran & Hilt, 2023; Mason et al., 2017).

Third, the cross-sectional design does not address questions regarding temporal sequence. Process oriented theoretical models suggest that greater interparental relationship discord leads to adolescent psychopathology (Schulz et al., 2005). However, there is also literature suggesting that offspring psychopathology can negatively influence parental relationship quality. For example, children between the middle childhood and teenage years are a frequently discussed topic of conflict between parents (Papp et al., 2009), and parents of a child with mental health difficulties may disagree about how best to parent, discipline, and care for their child. This disagreement and negativity can in turn negatively influence parental resources (i.e. the ability to engage in positive parenting behaviors), which can detriment the well-being of the entire family system (Hess, 2022). Additionally, childhood or adolescent emotional reactivity, a well-established transdiagnostic symptom of psychopathology, has been proposed as a mechanism by which offspring experience being “caught in the middle” (referred to as “triangulation”) of their parents’ conflict, which not only exacerbates their own experience of internalizing symptomatology, but may also inadvertently intensify their parents’ relationship distress (Buehler & Welsh, 2009).

Furthermore, a reciprocal, bidirectional influence of interparental relationship discord and adolescent psychopathology occurring over time is also possible, such as that found between interparental relationship discord and parents’ psychopathology (e.g., Whisman & Uebelacker, 2009). Although longitudinal associations between interparental relationship adjustment and offspring externalizing and internalizing behavior have been reported as similar in magnitude to cross-sectional associations (van Eldik et al., 2020), most of the studies included in this analysis examined children. Longitudinal studies of adolescents that were included in the same meta-analysis provide evidence that interparental relationship discord is associated with adolescent psychopathology over time. For example, Feinberg et al. (2007) found that interparental relationship discord at baseline was significantly associated with both adolescent depression and adolescent antisocial behavior three years later, and Cui et al. (2005) found that changes in interparental relationship discord over four annual assessments predicted changes over time in adolescent delinquency, substance use, and symptoms of anxiety, depression, and hostility. Additional longitudinal research is needed to examine the associations between interparental relationship discord and internalizing and externalizing psychopathology in adolescents over time.

Although observed associations between parental relationship discord and offspring psychopathology are often discussed in terms of possible influences on one another, it may be that there are other plausible alternative hypotheses, namely gene–environment correlations (rGE; Jaffee & Price, 2007). The genetic risk for psychopathology that is transmitted from parents to children may also lead to interparental relationship discord, which in turn leads to greater risk for child psychopathology (i.e., passive rGE) (Schermerhorn et al., 2011). For example, the personality trait of neuroticism has been shown to be highly heritable (Vukasović & Bratko, 2015), and high genetic loading for neuroticism that may be passed on from parents to offspring may increase risk for interparental relationship discord in parents (Heller et al., 2004) and psychopathology in their adolescent offspring (Castellanos-Ryan et al., 2016). Additional genetically informative designs, such as adoption studies or children of twins studies addressing competing mechanisms underlying the association between interparental relationship discord and adolescent psychopathology are needed.

In addition, genetically influenced adolescent psychopathology and corresponding behaviors may lead to greater interparental relationship discord (i.e., evocative rGE). One adoption study reported that child anger was positively associated with interparental conflict, and child pleasure was positively associated with interparental warmth (Ramos et al., 2022). Although several genetically informative studies have been conducted in the domain of interparental relationship functioning and adolescent psychopathology (Meyer et al., 2000; Schermerhorn et al., 2011), these studies have often examined single diagnoses or symptom clusters. A major limitation within this body of research is the absence of appropriate controls for potential confounds. Given the strength of behavioral genetic methods (i.e. co-twin and family-controlled designs) in accounting for variables that are difficult to measure, genetically informed designs are likely to be highly useful in this research area. Although a full review of the genetically informed research in this topic area is beyond the scope of the present study, additional genetically informed research is needed to distinguish between the potential causal influence of interparental relationship discord versus alternative hypotheses underlying this association for clinically elevated levels of psychopathology.

Despite the limitations and need for additional research outlined above, the present study remains novel and informative. Moreover, the present study has several unique strengths. First, a large probability sample of adolescents and their parents was examined. Second, well-established measures of interparental relationship discord and adolescent psychopathology were used. Third, because most of the diagnoses were based on adolescent report and interparental relationship discord was based on parent report, the observed associations cannot be attributed to single source bias.

If greater interparental relationship discord increases risk for a variety of manifestations of psychopathology in adolescents, targeted interventions aimed at improving discord may have far-reaching implications for the prevention and treatment of psychopathology in adolescents. Interventions that specifically target the interparental relationship in the context of intact households, divorce, or domestic violence have been shown to improve interparental relationship discord, reduce interparental conflict, improve communication and problem-solving, and increase coparenting, as well as improve child outcomes (for a review, see Harold & Sellers, 2018). From a clinical perspective, clinicians working with children and adolescents with specific disorders are faced with decisions regarding the selection and delivery of treatment. Understanding the nature of the association between interparental relationship discord and level of psychopathology in offspring that exceed diagnostic thresholds for severity, frequency, and degree of impairment may lend itself to the development of targeted clinical interventions aimed at improving outcomes, potentially mitigating cycles of intergenerational relationship functioning and psychopathology. Furthermore, research evaluating the associations between improvements in interparental relationship discord and change in mental health outcomes (i.e., decline in symptoms of psychopathology or no longer meeting diagnostic criteria for mental disorders) in offspring would be informative regarding the potential causal relationship between interparental relationship discord and adolescent psychopathology. Finally, results suggesting that adolescent psychopathology leads to interparental relationship discord would also have important implications for interventions. For example, evidence supporting this direction of effect might suggest that helping parents cope with adolescent psychopathology may help decrease interparental relationship discord.

## Data Availability

The data that support the findings of this study are available for public use. The NCS-A dataset can be found at: 10.3886/ICPSR28581.
